# External proficiency testing exercises: challenges and opportunities

**DOI:** 10.3389/fgene.2024.1304312

**Published:** 2024-02-09

**Authors:** Ilias Doxiadis, Claudia Lehmann

**Affiliations:** Institute for Transfusion Medicine, University Hospital of Leipzig, Leipzig, Germany

**Keywords:** external proficieny testing, histocompatibility and immunogenetics, HLA antibody screening, HLA, unacceptable antigens

Proficiency Testing (PT) provides the participant a certificate proving the competence and reliability of the laboratory. PT was very disputed before accreditation and certification of the laboratories performing diagnostic workout for patients. Historically, the report of the first two surveys of the Committee on Laboratories of the Medical society of the State of Pennsylvania of the year 1946 (Belk and Sunderman, 1947), revealed a for today’s standards unacceptable situation. For the determination of hemoglobin (37 satisfactory results vs. 35 unsatisfactory results) and for glucose (60 satisfactory results vs. 43 unsatisfactory results respectively). Honestly, these results were not only not adequate, but they also reflected the situation at that time. Similar findings were observed for almost all disciplines. These results promoted the use of PT on a primarily voluntarily basis. For Histocompatibility and Immunogenetics (H&I) PT was introduced during the International Histocompatibility Workshops to assure that the submitted data were reliable. Both the schemes of the International Cell Exchange (Lau et al., 1992) and the Eurotransplant Scheme (Doxiadis et al., 2000; Doxiadis and Claas, 2003) were introduced to improve the reliability of the participating laboratories. Especially in the field of organ transplantation, in which organs are offered and transported from center to center or from country to country according to the organ exchange organizations, the reliability of the laboratories is an imperative (Doxiadis et al., 2000). Furthermore, in stem cell transplantation the life of the patient relies on the accurate information from the H&I laboratory. PT is an integral part of the package a laboratory performing diagnostics must fulfill. At the beginning, the samples for PT were send out *ad hoc*. The laboratories received a small piece of the spleen from an organ donor, which was used for typing. The report of Schreuder et al. (1986) show that the results were far from adequate. They showed the efficacy and reliability of the participating centers during duty hours at that time. An increase of reliability from 40% in 1977 to 91% in 1981 was reported. Furthermore, Opelz et al. (1991) in 1992 showed that 25% of the reported HLA-DR serological results were incorrect when compared to molecular typing. A significant increase of reliability and efficacy of the laboratory in H&I was needed. Molecular typing was introduced in Eurotransplant and then worldwide. Interestingly, Sunderman (1992) the pioneer of PT mentioned that even “proficiency testing had its probable beginnings in the Paleolithic age … Neanderthal man tested his lethal stone axes for strength, weight, and serviceability before using them for the onslaught of his enemies”.

**FIGURE 1:**
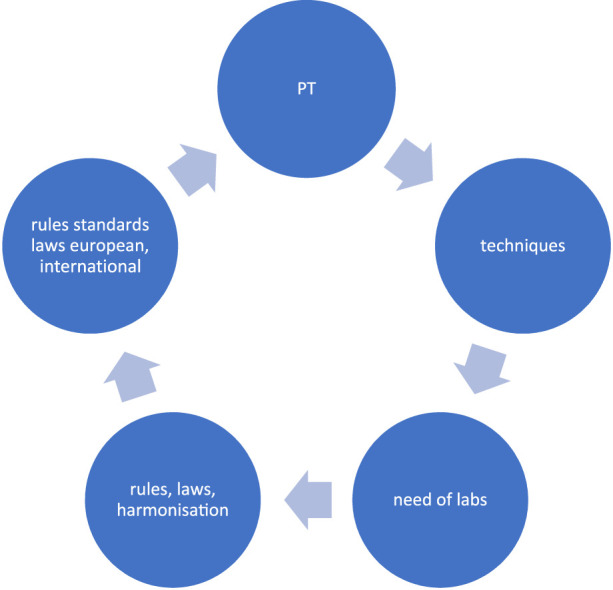
The evolution of PT.

In the beginning of the nineties of the past century, the schemes changed from an *ad hoc* manner to fixed dates. Exercises including the number of samples to be tested, their analyses, the way of calculation of discrepancies, the certificates, etc. were documented. This is mainly due to the PT Committee of the European Federation of Immunogenetics (EFI) and the respective Committees of the sister societies, like ASHI. The number of samples to be tested, and discrepancy calculation were institutionalized. A view how the evolution of PT occurs is presented in Figure 1. Here the influence of external factors is given. The report of the PT results changed with time. At the beginning the reports included the name of the laboratories, in an open way of reporting data. This had to be changed because of the privacy rules within the community. If this was a step in a good direction remains open. Nowadays laboratories and their services can be valued in social media. The main opportunity of the organizers is to cope with the daily work of the participants and promote flexibility. New methods as well as new ways of sample workouts ask for new schemes, but outdated methods should be stopped.

Among the evolution of PT is the digital input of the retrieved information. The digital input of the PT data and the information retrieved from PT is from the point of view of the organizers a major (evolutionary) step forward, because it helps the analysis and reduces as good as possible any clerical error from their part. However, this is not the case if the view of the participant is considered, who must enter the data mainly manually. Even if they follow the four eyes principle, meaning that two persons check the results prior to submit them, clerical errors can and will occur, following the saying: good lab but bad supervisor. This step must be automated in the future since it is one very important step for reliable PT results. In some countries the electronic patient file is or will be introduced soon, while PT organizers keep the manual entry of the results. To our opinion the entry of the data must follow the way results are reported to clinicians.

Besides Accreditation and Certification from the national bodies internal and external proficiency testing exercises are needed. Comparison of a laboratory to all the others is done in the modern times via professional European Institutions like NEQAS, ( *www.ukneqas.org.uk* ), Instand, ( *www.instand.ev.de* ), or the Eurotransplant Reference Laboratory ( *www.etrl.eurotransplant.org* ) and others. World-wide there are many institutions and laboratories organizing PT in a professional way on a scientific or diagnostic basis nationally or internationally, e.g., CAP, UCLA. The flow is similar for all organizers and participants. The participants apply for participations, the organizers inform the participants about the dates when the samples will be sent. The report of the results meets a deadline, and the results are sent to the organizer. A certificate of either participation or fulfil of the criteria set by the respective international or national society, e.g., APHIA, ASHI, EFI or others is issued. The certificate can be issued for every send out or annually.

Only when it was decided that accreditation/certification makes use of the results of PT to grant accreditation/certification, PT became mandatory. Furthermore, it received a status of “a must” for diagnostic purposes in H&I. All steps reported above led the laboratories to a diagnostic path with reliable results. PT must mimic the workflow laboratories perform their work. Furthermore, PT must be established for all diagnostic related testing performed in a laboratory. The possibility to receive accreditation without a PT is possible since the International Standard Organization (ISO) DIN ISO 15189, required for medical laboratories in diagnostics, offers the possibility to make use of intra laboratory control for the case no established PT is available. In the meantime, the different Societies follow this possibility. Within the Immunogenetical Societies (as ASHI, APHIA and EFI), in the past, the mixed lymphocyte culture assay and the T cell precursor assays could not be accredited because no PT was present or could be established. Laboratory comparisons are not specified in more detail, they are intended to test or compare what is required for practical application and are therefore a substitute or surrogate for an external PT. Among those assays, the monoclonal antibody immobilization of platelet antigens (MAIPA) assay is not offered by any provider, but an intra laboratory testing allows the possibility to receive an accreditation via ISO, as currently done in Germany. Other new techniques such as the Oxford Nanopore Technology (Liu, 2021), the modern absorption/elution method (Liwski et al., 2022) can be accredited without waiting long time until official PT are established. Similarly, the complement cytotoxicity assay (CDC) can be allowed in the future, since in some regions this assay is used for the final decision before transplantation, while in other regions this assay is not anymore performed.

The allocation of organs is influenced by the accuracy of the definition of HLA specific antibodies. Here, several methods can be used, with a high spectrum of sensitivity and reliability. Besides the complement dependent cytotoxicity (CDC), Luminex based assays can be used. Two different providers are available making the comparison of the results difficult (Israeli et al., 2015), furthermore, a new reader has been introduced, with an increased sensitivity, jeopardizing the comparison of the results. It is for no saying that the results of the screening for HLA specific antibodies influences allocation, transplantation, and post-transplant treatment of the patient. Currently, PTs have been established for these methods and are analyzed separately. Unacceptable HLA antigens defined as mismatches to be avoided in transplantation, are defined according to the results. To our opinion, PT organizers should concentrate on establishing PTs for the definition of such unacceptable HLA antigens, which in their turn are used in the virtual crossmatching procedure. A method-based analysis might offer valuable data, but it reflects more the ability of the participant to perform in comparison with others (Israeli et al., 2015), than the use of the results which directly influence a medical procedure. To our opinion there is here a strong discussion need. To our opinion specific calibration of the Luminex based assays and the readers might help in the comparison of the results. PT exercises are required for the medical procedures according to ISO 15189 such as definition of unacceptable antigens. To our observation the expertise within laboratories regarding the CDC assay is steadily decreasing, especially since the introduction of molecular typing techniques. In addition, CE-labelled commercial trays with frozen cells will be not available from 2025 on so that they cannot be used for diagnostic purposes in Europe. Reestablishing local test is quite cumbersome and difficult because of the *In-Vitro* Diagnostic Regulation (https://euivdr.com).

State-of-the-art laboratories in the meantime have introduced electronic storage of data, analyses of them, and reports to the clinicians. The main reasons are not only quickness, or storage of the results but especially prevention of man-made errors. This is one of the points needed for the state-of-the-art PT in the future. The report of the results of a PT to the organizer must be done electronically using reports in an up-to-date manner, as mentioned above, mainly using open but reliable protocols. This should be discussed between the PT organizer and the participants.

PT organizers must adhere to the changing requirements. Regarding the position of PT organizers in the complex situation within the different organizations and the accreditation/certification bodies we propose a direct contact and discussion which will lead to a reduced workload of the participants while reliability will increase. Till now the results of the PT exercises are regarded as an additive information for the performance of a PT participant. To our opinion the PT exercises must be used for future policies of the transplantation procedures within the National Bodies and Societies. One important aspect are the costs which should be optimal to reduce inconsistencies and allow access to PT world-wide.

In addition, PT exercises should be used nationwide for reimbursement from Medical Insurance Bodies. Here, Organizers covering laboratories Europe- or worldwide should be accepted by the National Medical Insurance Bodies, if the respective program is certified by the Societies or Organizations, EFI, ASHI, APHIA, etc.

In summary.

- PT is an integral step in an accreditation/certification procedure and is directly bound to the reliability of laboratory results.
- Entering the results electronically via reliable protocols and report the results to the accreditation bodies is an imperative.
- Use of the PT reports for accreditation/certification purposes must be done electronically to avoid unnecessary nature resources and costs
- Besides the “usual” PT, experimental PT could, and should be organized for new methodologies. In this case the Societies are asked to provide procedures
- All methods with no established PT should not be used for certification/accreditation unless interlaboratory prove the opposite
- The PT should keep the costs as low as possible to allow access for all laboratories. It is imperative to avoid unnecessary use of nature resources.
- PT providers need to evolve and develop their PT offering with the evolution of current/new laboratory techniques.
- The opportunities offered for PT organizers and participants are mainly in the flexible use of the programs. The modern view of PT allows short term schemes in which a few participants are taking part. Instead of waiting until a method is well established, experimental PT should be offered meeting the laboratory requirements for a good laboratory practice.
